# The Importance of Humanistic Approach to Traffic Accidents 

**Published:** 2019-08

**Authors:** Kamran B Lankarani

**Affiliations:** ^*a*^Health Policy Research center, Health Institute, Shiraz University of Medial Sciences, Iran.

## Abstract

Iran has witnessed one the fastest decline in the road traffic mortality in past decade. Despite this great achievement, the number of death toll has increased in past two years although in a small manner. The global situation is similarly alarming and recent figures show an increase in the number of fatalities and injuries despite the proclaimed goals for decade of action by the united nation. The current situation does not mean that there were negligence but it indicates more importantly the urgent need to combine the current engineering approach to more humanistic approach to this modern world preventable disaster. Risk perception insufficiency has been found to be a major driver of fatal events especially in younger victims. Although law enforcement and environmental support could initiate the change in behavior but there is need for a humanistic approach for persistent change in behavior.

**Keywords:**

Humanistic Approach, Traffic Accidents

